# Structure-Function Investigation of Vsp Serotypes of the Spirochete *Borrelia hermsii*

**DOI:** 10.1371/journal.pone.0007597

**Published:** 2009-10-30

**Authors:** Rohit Mehra, Diana Londoño, Marie Sondey, Catherine Lawson, Diego Cadavid

**Affiliations:** 1 Department of Neurology, Neuroscience and Center for Emerging Pathogens at UMDNJ-New Jersey Medical School, Newark, New Jersey, United States of America; 2 Center for Immunology and Inflammatory Diseases, Massachusetts General Hospital, Charlestown, Massachusetts, United States of America; 3 Department of Chemistry and Chemical Biology, Rutgers University, Piscataway, New Jersey, United States of America; University of California Merced, United States of America

## Abstract

**Background:**

Relapsing fever (RF) spirochetes are notable for multiphasic antigenic variation of polymorphic outer membrane lipoproteins, a phenomenon responsible for immune evasion. An additional role in tissue localization is suggested by the finding that isogenic serotypes 1 (Bt1) and 2 (Bt2) of the RF spirochete *Borrelia turicatae*, which differ only in the Vsp they express, exhibit marked differences in clinical disease severity and tissue localization during infection.

**Methodology/Principal Findings:**

Here we used known *vsp* DNA sequences encoding for *B. turicatae* and *Borrelia hermsii* Vsp proteins with variable regions and then studied whether there are differences in disease expression and tissue localization of their corresponding serotypes during mouse infection. For sequence and structural comparisons we focused exclusively on amino acid residues predicted to project away from the spirochetes surface, referred to as the Vsp dome. Disease severity and tissue localization were studied during persistent infection with individual or mixed serotypes in SCID mice. The results showed that all Vsp domes clustered into 3 main trunks, with the domes for *B. turicatae* Vsp1 (BtVsp1) and BtVsp2 clustering into separate ones. *B. hermsii* serotypes whose Vsp domes clustered with the BtVsp1 dome were less virulent but localized to the brain more. The BtVsp2 dome was the oddball among all and Bt2 was the only serotype that caused severe arthritis.

**Conclusion/Significance:**

These findings indicate that there is significant variability in Vsp dome structure, disease severity, and tissue localization among serotypes of *B. hermsii*.

## Introduction

Relapsing fever (RF) is an arthropod-borne, spirochetal disease of humans caused by infection with different *Borrelia* species [1]. A characteristic feature of RF is two or more periods of high fever and bacteremia separated by afebrile periods during low bacteremia. This pattern of relapses and remissions is explained by the sequential spontaneous appearance and clearance of isogenic serotypes that are antigenically distinct from the previous ones and from the ones that follow them [1], [2]. Major outer membrane lipoproteins that are highly variable in sequence and are the target of antibody-mediated clearance define the serotype [3], [4]. These proteins in the new world RF spirochetes *B. hermsii* (Bh) and *B. turicatae* (Bt) come in two sizes, variable small proteins (Vsp) of ∼22 kDa and variable large proteins (Vlp) of ∼37 kDa [5], [6]. By spontaneously switching expression at a single expression locus by gene conversion from silent genes RF spirochetes evade antibody-mediated clearance [2]. Studies in our laboratory indicate that another consequence of serotype switch is changes in disease severity and tissue localization during infection: Serotype 1 of *B. turicatae* (Bt1), defined by expression of Vsp1, localizes to the brain in 5- to 10-fold higher numbers than serotype 2 (Bt2), defined by expression of Vsp2 [7], [8], [9], [10]. Conversely, Bt2 infection features 5- to 10-fold higher pathogen load in the blood, joints, heart, and skin than Bt1 [11], [12]. Furthermore, Bt2 causes more severe systemic disease than Bt1, including conjunctivitis, ruffled skin, tibiotarsal arthritis, reduced spontaneous activity, and neonatal mortality [7], [8], [9], [10], [12], [13], [14], [15], [16], [17].

There have been no previous studies of the virulence and tropism of isogenic serotypes in *B. hermsii*. However, the previous sequencing of all *vsp* genes from *B. hermsii* strain HS1 by Barbour and colleagues [5], [18] gave us the opportunity to study whether differences similar to what we observed with B. turicatae do exist with *B. hermsii*. Furthermore, because of the recent characterization of crystal structures from Vsp1 of *B. turicatae* [19], [20] and OspC from *B. burgdorferi* [21] we were able to study the heterogeneity of the Vsp variable dome region using computer homology modeling. The results revealed significant heterogeneity in the Vsp dome region and in the virulence and tissue localization of *B. hermsii* serotypes.

## Methods

### Bacterial strains

*B. hermsii* strain HS1 [22] and *B. turicatae* Oz1 strain [8] were used for all experiments. The identity of *B.hermsii* serotypes 3 and 13 was confirmed by PCR amplification and sequencing of the expressed *vsp* gene from spirochetes cultured from infected tissues [22]. The identity of Bt1 and Bt2 was determined by immunoblot with anti Vsp1 or Vsp2 monoclonal antibodies as before [23], [24]. All borrelias were cultured in BSK-H media supplemented with 6% rabbit serum (Sigma). Peak bacteremia and spirochetal viability were determined using phase-contrast microscopy with a Petroff-Hauser chamber [25].

### Mouse Infections

All mice used in these experiments were 4–5 week old female CB17-SCID (severe combined immunodeficiency) or *Balb/c* mice (Charles River). Mice were inoculated intraperitoneally with a total of 10^3^ viable spirochetes from BSK-H cultures or pooled necropsy plasma suspended in 200 µL of PBS. Mice sham-inoculated with PBS or with a non-infectious *B. burgdorferi* B31 derivative [26] were used as negative controls. All mice were maintained in a germ free environment and housed in accordance with the Animal Welfare Act in facilities accredited by the AAALAC (at UMDNJ-New Jersey Medical School). To generate a diverse mixture of *B. hermsii* serotypes we inoculated immunocompetent *Balb/c* mice (N = 3) with 500 spirochetes each of Bh7 and Bh19 (a kind gift from Dr. Alan G. Barbour, UC Irvine, CA) and 7 days later harvested the blood containing the first relapse serotypes from each mice, pooled it, and amplified it by inoculation into SCID mice (N = 3) to grow to peak bacteremia without negative selection from serotype-specific antibodies. This amplified population, referred to as R1, was then inoculated into a new group of *Balb/c* mice (N = 3) and 7 days later the process was repeated by collecting the blood from each mouse, pooling it, and inoculating it back into new SCID mice to grow to peak bacteremia. The process was similarly repeated with this second group of relapse serotypes, referred to as R2. A total of 5 such cycles of negative selection in *Balb/c* mice followed by amplification in SCID mice were completed, followed by a final amplification step of one each of all the R1 to R5 aliquots into a final group of 4 SCID mice. Pooled plasma from these 4 SCID mice, referred to as relapse mix, was predicted to contain a highly diverse mixture of relapse serotypes; it was aliquoted and frozen in 10% DMSO for later use.

### Clinical examination

All mice were examined by an examiner (MS) masked to infection status and serotype. Severity of clinical disease was assessed upon disease onset every 3–4 days for 2 weeks using the following semi-quantitative clinical score: (A) Skin (fur): normal = 0; ruffled = 1; ruffled and dry = 2. (B) Eyes (conjunctival secretion): normal = 0; mucous = 1; mucous and closing of eyelids = 2. (C) Spontaneous activity: normal = 0; reduced = 1; very little/absent = 2. (D) Tibiotarsal joints: normal = 0; swollen = 1; swollen and red = 2. The severity of arthritis was also measured quantitatively on the most swollen tibiotarsal joint with a Vernier caliper [8].

### Tissue and Fluid Collection

Mice were euthanized by inhalation of isoflurane. Heparin was used as an anticoagulant for blood collection by heart puncture. Necropsy plasma was obtained by whole-blood centrifugation for 5 sec in a tabletop centrifuge. Total body perfusion with 30 mL PBS followed by rinsing of the brain with 1 mL PBS twice in sterile 2-mL microfuge tubes (Sarstedt Inc, Newton, NC) were used to minimize residual blood contamination of the brain [22]. The whole brain was homogenized in sterile PBS using plungers of sterile, 1-mL plastic syringes (Becton Dickinson & Co., Mountain View, CA) followed by further homogenization using glass microbeads (Lysing Matrix D, MP Biomedicals, Irvine CA) with the FastPrep-24® system at 4.0 M/s for 30 seconds twice (MP Biomedicals, Irvine, CA) and stored frozen at −80°C for DNA extraction later on. Pellets of necropsy plasma were prepared by centrifugation at 13,000 rpm for 20 min at room temperature.

### DNA extraction and TaqMan PCR

DNA was extracted from volumes of brain homogenates equivalent to 10 mg of brain or from pellets of whole necropsy plasma using the QIAamp DNA Micro® kit (Qiagen Inc., Turnberry, CA), eluted in 100 µL TE, and kept frozen at −80°C. TaqMan PCR reaction used 100 ng of brain or 10 ng of plasma pellet DNA. TaqMan assays were run in multiplex, with the housekeeping gene and target gene in the same well, and in singleplex, with the housekeeping gene and target gene in different wells, with pre-designed mouse 18S rRNA primers and probe (Applied Biosystems Mm4308329) to control for the amount of host input DNA. Custom designed primers and probes for the *B. hermsii* chromosomal *rrs* gene for 16S ribosomal RNA and the linear plasmid genes *vsp3*, *vsp13*, *vsp2 and vsp27* were made by Applied Biosystems (Table 1). We calculated the ΔΔCt number by subtracting the Ct for each *vsp* gene from the Ct for the *16S rRNA* borrelial chromosomal gene and adjusted the results for the Ct value of the mouse housekeeping gene *18S rRNA*. When no mouse 18S rRNA signal was obtained, as was the case in some plasma pellet samples, a Ct of 40 was used. The sensitivity to detect any of the *Bhvsp* genes in brain decreased from one and a half to almost 4 fold when the samples were run in multiplex compared to singleplex because of the abundance of host relative to borrelial DNA. All samples were run in triplicate as relative quantification assays on the ABI Prism 7500 real time PCR system (Applied Biosystems, Alameda, CA). Samples with H_2_O instead of DNA and DNA from uninfected mice brains (only for experiments with borrelia genes) were used as negative controls.

**Table 1:** Taqman primers and probe sets used for relative quantification of *B. hermsii* genes.

16S *rRNA*	
Forward Primer	5′-GCGTAAAATAC CACAGCTCAACTG-3′
Reverse Primer	5′-CCCCTATCAGAC TCTAGTCATGCA-3′
Probe^a^	5′-TTTCCAGC ATATTCCC-3′
*vsp3*	
Forward Primer	5′-AGTGAAGCATTC GTAACCCAAGTAA-3′
Reverse Primer	5′-CATGTGCATCA GTAACACCTTCTTT-3′
Probe	5′-ATCAAAGCAT ACTGATCTTGC-3′
*vsp13*	
Forward Primer	5′-CAAGTTGTTGCTA TTAAGACTGCAAGT-3′
Reverse Primer	5′-GCATCGTCATTAC TAACCTCGTTTT-3′
Probe	5′-CCAAGTTCA GCATTTTT-3′
*vsp2*	
Forward Primer	5′-ATGTAGGTACTAAATTGGACGGGTTA-3′
Reverse Primer	5′-TTGCTTAAGAATGCTGTACCTTTACTCT-3′
Probe	5′-TGGAATTTCTGAAGATATTAAG-3′
*vsp27*	
Forward Primer	5′-GGTATTTAGTGTGGCTACAACTATAGA-3′
Reverse Primer	5′-GCCTTAGCCTCAACATCTTTAACTTT-3′
Probe	5-ACAAGTAGCACAATCTC-3′

a All probes were FAM-labeled; for the origin of the *vsp* primers and probes see Table S1 and references [5] for the *vsp* and [46] for the *16S rRNA* genes.

### Computer structural homology modeling of Vsp domes

Computer homology models were prepared for Vsps corresponding to several Vsp serotypes from *B. hermsii* HS1 and *B. turicatae* Oz1 strains. For this, five known crystallographic source structures were used, including three different *B. burgdorferi* OspC variants (B31, N40, HB19) [21], [27] and *B. turicatae* Vsp1 in two different crystal forms [19], [20]. The Protein Databank ids for these structures are 1yjg and 2ga0 for BtVsp1, 1g5z for Bb N40 OspC, 1f1m for Bb HB19 OspC, and 1ggq for Bb B31 OspC. All five structures are defined to near-atomic resolution (1.8–2.7 Å) and all have reasonable model statistics and geometry (R_free_ 21.5–27.5%, r.m.s. bonds 0.004–0.022 Å, no Ramachandran plot outliers). The initial step in the homology modeling process was careful preparation of a multiple sequence alignment of each Vsp target with the crystal structures, guided by inspection of the superimposed models using molecular graphics. Within variable loops, gaps and/or insertions were positioned to maximize similarity of residue substitutions. For example, introduction of staggered gaps into the alignment between BtVsp1 and BtVsp2 better conserves hydrophobic burial properties of a variable loop between helices 2 and 3. The multiple sequence alignments and single-subunit structural models were the inputs to automated homology modeling using the program Modeller [28] and/or to the Swiss Model server [29]. Full Vsp dimers were then generated from single subunit coordinate sets by application of two-fold symmetry; this procedure is simpler than generating full dimer homology models. All model dimers were inspected for problem areas (Ramachandran outliers, high energy areas, poor geometry areas, clashes at the dimer interface). Problems were corrected either by revision of the multiple sequence alignment, or by manual adjustments of the model, guided by the crystal structures. Model coordinates were subsequently optimized with respect to standard geometry [30] and constrained to obey perfect two-fold symmetry using Refmac without X-ray restraints [31]. The homology models lacked only the N-terminal and C-terminal residues that are not present in the crystal structures. Pairwise alignment scores of Vsp dome region sequences were obtained using ClustalW (Version 7.6.0.87). All known Vsp proteins from *B. hermsii* HS1 and *B. turicatae* Oz1 Vsp's were included in this analysis (Table S1).

### Statistical analyses

All statistical analyses used GraphPad PRISM Version 5.01. To compare the amounts of *vsp* DNA in the brain and blood we used one way ANOVA with Bonferroni's multiple comparison tests. To compare peak bacteremia in necropsy blood we used t-tests. We also used t-tests to compare the severity of clinical scores among individual borrelia serotypes. The error bars represent standard deviations. P values ≤0.05 were considered significant.

## Results

### Computer homology modeling of Vsp dome regions

**Figure 1:**
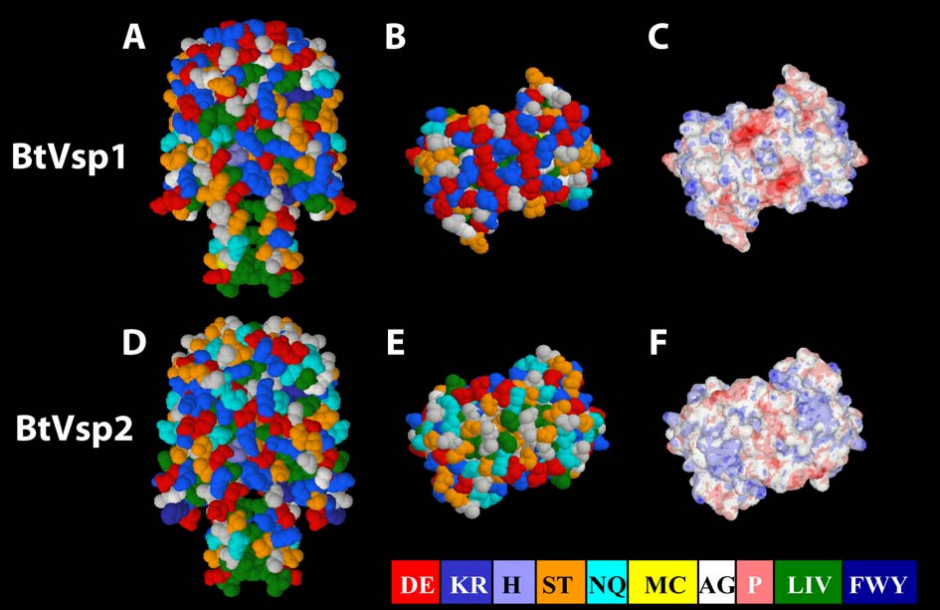
Computer homology modeling of *B. turicatae* Vsp1 (BtVsp1) and Vsp2 (BtVsp2). Vsp variable dome regions were modeled based on two crystal structures of BtVsp1 and OspC from 3 different *Borrelia burgdorferi* strains. Panels A&D show the sagital views, panels B&E the axial views, and panels C&F the electrostatic potential field of the axial view. Each amino acid corresponds to a letter and a color which represents their polarity with red for high polarity and dark blue for low polarity. Notice the dome of BtVsp1 is much more polar and its negative electrostatic potential is more pronounced than that of BtVsp2 (D = aspartic acid, E = glutamic acid, K = lysine, R = arginine, H = histidine, S = serine, T = threonine, N = asparagine, Q = glutamine, A = alanine, G = glycine, P = proline, L = leucine, I = isoleucine, V = valine).

We began the study by examining the differences between Vsp1 (BtVsp1) and Vsp2 (BtVsp2) of *B. turicatae* by computer homology modeling. For this we predicted the structure of BtVsp2 using its known DNA sequence based on 5 known crystal structures from Vsp and OspC proteins [32], two from *B. turicatae* Vsp1 (BtVsp1), and 3 from *Borrelia burgdorferi* OspC [21], [33]. A comparison of the generated BtVsp2 structure with that of BtVsp1 revealed striking differences restricted to the portion that projects away from the spirochetal surface, referred to as the Vsp dome (Figure 1). Although both domes are hydrophilic, the BtVsp2 dome has many fewer charged residues. As a result, the BtVsp1 dome has higher polarity and a more negative electrostatic potential.

### Cluster analysis of Vsp domes from *B. turicatae* and *B. hermsii*

**Figure 2:**
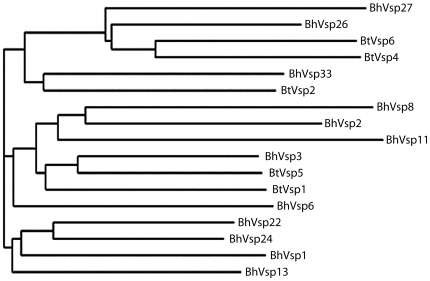
Phylogenetic tree of the Variable small protein (Vsp) “dome” region from 12 *B. hermsii* (Bh) and 5 *B. turicatae* (Bt) *serotypes*. The tree is based on sequence alignment of amino acids from the dome Vsp region, defined by residues 67–183 in BtVsp1 (see materials and methods for details and Table S1) using ClustalW. Three main branches were identified with BtVsp1 clustering in the middle branch and BtVsp2 in the upper branch.

Because the differences between BtVsp1 and BtVsp2 proteins is in their dome region, next we did a cluster analysis of the dome region from all known Vsp proteins from *B. hermsii* (N = 12) and *B. turicatae* (N = 5) using the ClustalW algorithm (Table S1) that treats multiple alignments like single sequences and compares them in a two by two progression to cluster them by similarity. The resultant dendrogram showed that all Vsp domes clustered into 3 branches, with the BtVsp2 in the upper branch and the BtVsp1 dome in the middle branch (Figure 2). We also calculated pairwise alignment scores of the discontinuous sequences that constitute the dome regions of BtVsp1 and BtVsp2 for all 12 known Vsp proteins from *B. hermsii*. This revealed that BhVsp3 and BhVsp24 were the *B. hermsii* Vsp domes most similar to the BtVsp1 dome, with scores of 48 and 45, respectively (Table 2). The BhVsp3 dome was more similar to the BtVsp1 dome than to the BtVsp2 dome, as reflected by a gap of 8 points between the two scores. The BhVsp13 dome had the highest pairwise alignment score with the BtVsp2 dome, 48 points, but only a gap of 4 points with the BtVsp1 dome. This indicated that the BhVsp3 dome was more similar to the BtVsp1 dome than the BhVsp13 dome was to the BtVsp2 dome.

**Table 2:** Pairwise alignment scores of the discontinuous sequences that constitute the dome regions of all known *B. hermsii* Vsp's with Vsp1 (BtVsp1) and Vsp2 (BtVsp2) of *B. turicatae*.

*B. hermsii*Vsp	*B. turicatae* Vsp		Score Gap
	BtVsp1	BtVsp2	BtVsp1-BtVsp2
BhVsp1	38	44	−6
BhVsp2	43	41	2
BhVsp3	48	40	8
BhVsp6	40	39	1
BhVsp8	39	36	3
BhVsp11	37	30	7
BhVsp13	44	48	−4
BhVsp22	44	46	−2
BhVsp24	45	42	3
BhVsp26	43	42	1
BhVsp27	33	40	−7
BhVsp33	42	46	−4

### Differences in clinical disease expression among *B.hermsii* serotypes

**Figure 3:**
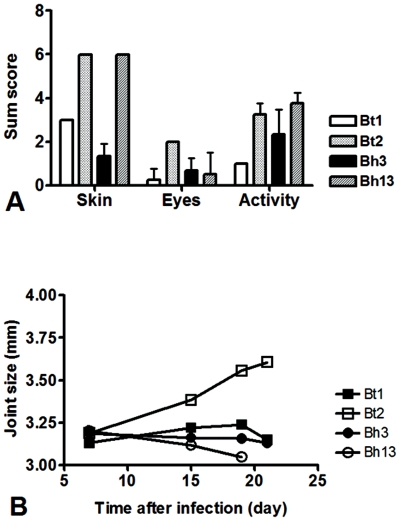
Severity of clinical disease in SCID mice persistently infected with isogenic serotypes of *B. turicatae* (Bt1 or Bt2) or *B. hermsii* (Bh3 or Bh13). Mean (SD) sum clinical severity scores (panel A) and tibiotarsal joint swelling measured by a Vernier caliper (in mm, panel B) in groups of 4 SCID mice each studied by a masked examiner every 3–4 days for 3 weeks during infection with serotypes 1 (Bt1) or 2 (Bt2) of *B. turicatae* or serotypes 3 (Bh3) or 13 (Bh13) of *B. hermsii*. Notice greater disease severity scores and tibiotarsal joint swelling in mice infected with Bt2. Also notice the greater similarities between Bt1 and Bh3 and between Bt2 and Bh13. Table 3 shows the p values for the paired comparisons among individual serotypes.

We selected the two serotypes of *B. hermsii* strain HS1 whose Vsp domes had the highest pairwise alignment scores compared with BtVsp1 and BtVsp2, *B. hermsii* serotypes 3 (Bh3) and 13 (Bh13), respectively (Table 2), to study their virulence and tissue dissemination during infection in comparison to Bt1 and Bt2. For this, groups of four 4–5 week old female CB17-SCID mice were inoculated intraperitoneally with 10^3^ spirochetes of each selected serotype and examined clinically every 3–4 days for 3 weeks by a masked observer (MS). Mice inoculated with a non-infectious derivative of *B. burgdorferi* B31 [26] was included as negative control. Clinical disease severity was scored semiquantitatively for skin, eyes, joints, and spontaneous activity, and quantitatively for tibiotarsal joint swelling using a vernier caliper [8]. The results revealed that mice inoculated with Bh3 had similar disease severity scores than mice inoculated with Bt1 (Figure 3A). In contrast, mice inoculated with Bh13 had disease severity scores for skin and spontaneous activity that were more similar to Bt2. Interestingly, Bt2 was the only serotype that caused prominent tibiotarsal joint swelling (Figure 3B). None of the mice that were inoculated with the non-infectious B31 derivative developed any signs of disease. Paired comparisons of disease severity scores among the 4 serotypes showed that Bt2 was the most virulent, resulting in significantly worse disease scores for all 4 clinical measures compared to Bt1, for 3 of the 4 measures compared with Bh3, and for 2 of 4 measures compared to Bh13 (Table 3). Bh13 was the second most virulent with worse clinical disease severity scores in 2 out of 4 measures compared to Bt1 or Bh3. Bt1 and Bh3 were the most similar among them. We concluded that, similar to *B. turicatae*, there are differences in the systemic virulence of *B. hermsii* serotypes.

**Table 3:** Paired comparison of clinical severity scores between groups of SCID mice persistently infected with serotypes 1 (Bt1) or 2 (Bt2) of Borrelia turicatae or serotypes 3 (Bh3) or 13 (Bh13) of *Borrelia. hermsii* a.

Clinical Involvement	Bt2 vs Bt1	Bt2 vs Bh3	Bt2 vs Bh13	Bt1 vs Bh3	Bh13 vs Bt1	Bh13 vs Bh3
Skin	Bt2>Bt1	Bt2>Bh3	Bt2 = Bh13	Bt1>Bh3	Bh13>Bt1	Bh13>Bh3
p value^b^	0.008	0.018	1	0.018	0.008	0.018
Eyes	Bt2>Bt1	Bt2>Bh3	Bt2>Bh13	Bt1 = Bh3	Bh13 = Bt1	Bh13 = Bh3
p value	0.011	0.018	0.04	0.31	0.85	0.5
Joints	Bt2>Bt1	Bt2>Bh3	Bt2>Bh13	Bt1 = Bh3	Bh13 = Bt1	Bh13 = Bh3
p value	0.017	0.029	0.013	1	0.32	0.25
Spontaneous activity	Bt2>Bt1	Bt2 = Bh3	Bt2 = Bh13	Bt1 = Bh3	Bh13>Bt1	Bh13>Bh3
p value	0.011	0.19	0.18	0.07	0.011	0.05

a Groups of 4 SCID mice each were inoculated intraperitoneally with 10^3^ spirochetes of each serotype and upon onset of clinical disease examined on 4 different occasions over a 3 week period by a masked examiner to measure disease severity scores in the skin, eyes, joints, and spontaneous activity. A group of uninfected mice was included as negative control (not shown). The sign “>” indicates significantly higher disease severity score with the corresponding p value shown below.

b *P* value for the difference in mean (SD) severity scores for each clinical measure between each pair of serotypes shown above (t-test).

### Differences in peak bacteremia with *B. hermsii* serotypes

Next we studied whether there were differences in peak bacteremia among *B. hermsii* serotypes. For this we measured peak bacteremia during persistent infection [34] in SCID mice 3 weeks after inoculation with Bh3 or Bh13 and compared them with SCID mice similarly inoculated with Bt1 or Bt2. Bacteremia was measured using phase-contrast microscopy of tail vein blood with a Petroff-Hausser chamber. The results showed that Bh13 caused higher peak bacteremia than Bh3: their mean (SD) peak bacteremia per ml was 4.5×10^7^ (1.22×10^7^) and 1.67×10^7^ (1.04×10^7^), respectively (p<0.05). Consistent with previous results, Bt2 also caused higher mean (SD) peak bacteremia per ml than Bt1, 8.25×10^7^ (4.87×10^7^) versus 8.75×10^6^ (2.5×10^6^), respectively (p<0.05). Overall there was no difference in peak bacteremia between *B. hermsii* and *B. turicatae* when both serotypes were considered together (p = 0.54). We concluded that, similar to *B. turicatae*, peak bacteremia differs among *B. hermsii* serotypes.

### Differences in dissemination to the brain among *B.hermsii* serotypes

Next we compared the ability of *B. hermsii* serotypes to infect the brain. For this we wanted to include serotypes representing all three branches from the Vsp dome homology dendrogram (Figure 2). However, we could not get clonal populations from any Bh serotypes from the upper branch. As an alternative approach, we inoculated into the same group of SCID mice (N = 8) a diverse mixture of *B. hermsii* serotypes that we had prepared by serial passage of relapse serotypes into immunocompetent mice (see methods for details). Unlike the previous experiments of SCID mice inoculated with individual serotypes, this novel approach required a method capable of differentiating multiple serotypes simultaneously present in each mouse. For this we first tried standard PCR amplification of the expressed *vsp* gene that uses a forward primer specific for the *vsp* promoter and reverse primers specific for conserved regions near the 3′ of *vsp* genes [22]. However, the results were inconsistent and of low yield, especially in the brain. As an alternative we turned to TaqMan PCR because of its superior sensitivity due to a shorter length of the amplicons (<80 bp) relative to expressed *vsp* gene PCR (>1 Kbp). However, a limitation of the *vsp* TaqMan approach was that all serotypes carry both silent and expressed *vsp* genes and therefore any *vsp* TaqMan primers and probe set will measure spirochetes independently of the *vsp* gene being expressed. To address this limitation we took advantage of the fact that each serotype carries at least one extra copy of the *vsp* gene being expressed [35]. Accordingly, each serotype is expected to carry a higher *vsp* gene copy number when tested with TaqMan primers and probe sets specific for their expressed *vsp* gene than when examined with primer and probe sets specific for non-expressed, or silent, *vsp* genes. For this we prepared TaqMan primers and probe sets specific for *B. hermsii vsp2*, *vsp3*, *vsp13*, and *vsp 27*, corresponding to serotypes from all 3 branches of the Vsp homology dome dendrogram (Figure 2). All custom made TaqMan primers and probes were found to have high amplification efficiency when run either as singleplex or multiplex with the mouse housekeeping gene *18S rRNA* (Table 4). In most cases they turned out to be highly specific because they did not amplify DNA from *B. turicatae* (Table S2) or *B. burgdorferi* B31 (not shown). We demonstrated the validity of the *vsp* TaqMan approach by measuring blood and tissue samples from SCID mice that had been inoculated with Bh13 using *vsp13* and *vsp3* TaqMan sets. The results showed that the *vsp* gene copy number was twice as much with the *vsp13* than with the *vsp3* primers and probe set (Table S3).

**Table 4:** Efficiency of Taqman real time PCR Gene Expression Assays used in the study.

Assay	Taqman assay	Mean Efficiency	95% Confidence Interval
Borrelia 16S rRNA	Singleplex	98.10%	96.3%–99.8%
*Bhvsp3*	Singleplex	99.00%	88.3%–109.8%
*Bhvsp13*	Singleplex	100.00%	95.9%–104.1%
*Bhvsp2*	Singleplex	101.10%	97.8%–104.0%
*Bhvsp27*	Singleplex	99.00%	95.4%–100.9%
Borrelia 16S rRNA	Multiplex^a^	96.10%	88.5%–103.7%
*Bhvsp3*	Multiplex	97.80%	88.2%–107.3%
*Bhvsp13*	Multiplex	96.30%	86.7%–105.8%
*Bhvsp2*	Multiplex	99.00%	95.8%–102.0%
*Bhvsp27*	Multiplex	98.60%	96.6%–100.6%

a Multiplex refers to simultaneous amplification of a *Borrelia* gene and the mouse housekeeping gene (*18S rRNA*) in the same well.

**Figure 4:**
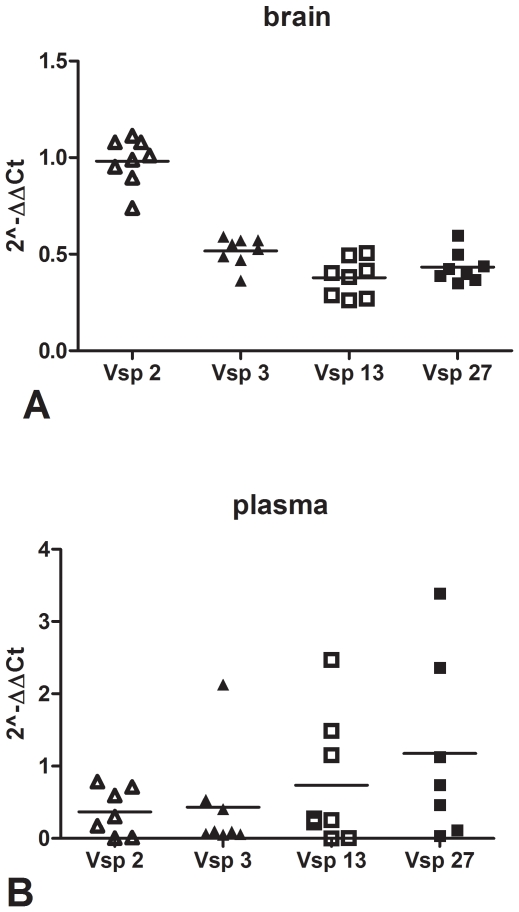
*B. hermsii vsp* gene copy number in brain and blood. The ability of serotypes 2, 3, 13, and 27 of *B. hermsii* to disseminate to the brain was examined by measuring the relative *vsp* gene copy number by TaqMan PCR in brain and plasma of SCID mice 2 weeks after intraperitoneal inoculation with a mixed relapse serotype population (see methods for details). Results were analyzed using the ΔΔCt method adjusting for the amount of input DNA using the mouse chromosomal gene for *18S rRNA* and graphed as the log 2 negative number (2^−ΔΔCt^) so that higher values present higher gene copy number relative to the borrelial chromosomal gene for *16S rRNA*. Results shown for plasma represent multiplex and for brain singleplex because the sensitivity for detection of borrelial genes in the brain is 1.5–3.6 fold lower with multiplex due to the abundance of host DNA. There was significant variability in gene copy number in brain (panel A, p<0.01 by one way ANOVA) but not in plasma (panel B, p = 0.47). Notice the higher gene copy number in the brain for *Bhvsp2* compared to *Bhvsp3*, *Bhvsp13*, or *Bhvsp27*, and for *Bhvsp3* compared to *Bhvsp13* (all p<0.05 after Bonferroni's correction).

We then used the *vsp* TaqMan approach to compare the relative abundance of *vsp2*, *vsp3*, *vsp13*, and *vsp27* gene copy number in the “relapse mix” inoculum (see methods for details). The results showed differences among the 4 *vsp* genes, with the highest gene copy number for *vsp3* and the lowest for *vsp2* (Table S2). We then proceeded to use the *vsp* TaqMan approach to compare the relative neuroinvasiveness of these 4 *B. hermsii* serotypes. For this we used blood and brain from 8 SCID mice that had been inoculated intraperitoneally with 10^4^ spirochetes of the “relapse mix” inoculum and necropsied 2 weeks later. The results were analyzed using the ΔΔCt method [36] which controls for the amount of host input DNA and expressed as the mean (SD) log 2 negative number (2^−ΔΔCt^). This analysis revealed significant differences in the *vsp* gene copy number between these 4 serotypes in the brain (Figure 4a, p<0.01 by one way ANOVA) but not in the blood (p = 0.47, Figure 4b). The comparison of pairs of *vsp* gene copy numbers in the brain showed more *vsp2* than *vsp3*, *vsp13* or *vsp27*, and more *vsp3* than *vsp13* (all p<0.05 using Bonferroni's correction). There appeared to be more *vsp13* than *vsp3* gene copy number in the plasma (Figure 4b), although the difference did not reach statistical significance: the mean (SD) 2^−ΔΔCt^ was 0.73(0.88) for *vsp13* and 0.43(0.7) for *vsp3*. This could be explained in part by the higher amount of Bh3 than Bh13 spirochetes in the original inoculum (Table S3). We did not find any correlation between the *vsp* gene copy number in the brain and the plasma (correlation coefficient = 0.10). These results revealed that the two *B. hermsii vsp* genes corresponding to serotypes from the middle branch of the Vsp homology dendrogram, BhVsp2 and BhVsp3, showed greater localization to the brain than the two belonging to the upper (BhVsp27) or lower (BhVsp13) branches.

### Computer homology modeling of *B. hermsii* Vsp domes

**Figure 5:**
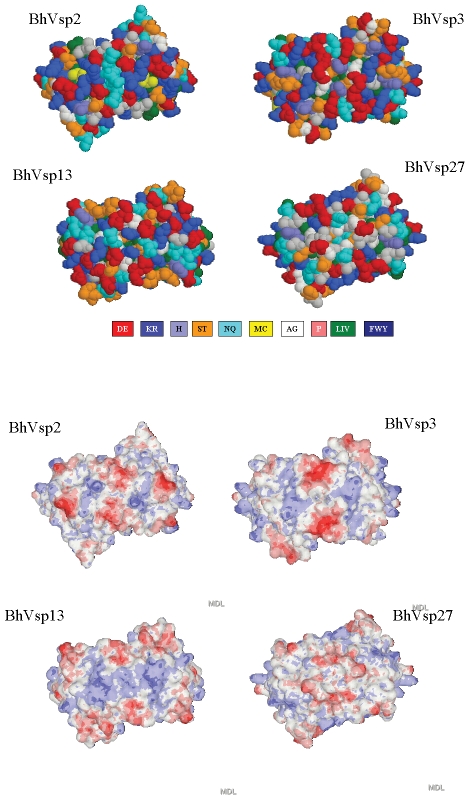
Computer homology modeling of *B. hermsii* Vsp's. Vsp dome regions were modeled based on two crystal structures of BtVsp1 and individual crystal structures from OspC from 3 different *Borrelia burgdorferi* strains. The upper panel shows the axial (dome) views of the Vsp proteins for *B. hermsii* serotypes 2 (BhVsp2), 3 (BhVsp3), 13 (BhVsp13), and 27 (BhVsp27). Each amino acid corresponds to a letter and a color which represents their polarity with red for high polarity and dark blue for low polarity. The lower panel shows the corresponding electrostatic potential fields for each axial region with the red color representing negative and the blue color positive electrostatic potential. Notice the more pronounced negative electrostatic potential of BhVsp2 and BhVsp3 (D = aspartic acid, E = glutamic acid, K = lysine, R = arginine, H = histidine, S = serine, T = threonine, N = asparagine, Q = glutamine, A = alanine, G = glycine, P = proline, L = leucine, I = isoleucine, V = valine).

Next we compared the structure and electrostatic field potential of the Vsp domes from Bh2, Bh3, Bh13, and Bh27. For this we did computer homology modeling as before using the known crystal structures of Vsp1 of *B. turicatae* and OspC from *B. burgorferi*. The results revealed that all four *B. hermsii* Vsp domes are more similar to *B. turicatae* Vsp1 than to *B. turicatae* Vsp2 (Figure 5, upper panel). The *B. hermsii* Vsp modeling also revealed that all 4 have multiple sites capable of participating in chelation of ions near their top, including a negative charge patch in electrostatic potential plus the presence of Asp, His or Cys residues (Figure 5, lower panel).

## Discussion

Here we studied using a murine model whether serotypes of the RF spirochete *B. hermsii* exhibit differences in their pathogenicity and pathogen load in blood and brain. The results revealed that, similar to what has been observed for the RF spirochete *B. turicatae*, for most aspects of clinical disease severity, peak bacteremia, and dissemination to the brain this was in deed the case. Although in most aspects the clinical manifestations of infection with *B. hermsii* serotypes resembled those observed with *B. turicatae*, one notable exception was tibiotarsal arthritis which occurs to a significant degree only with Bt2 (Figure 3B and Table 3). In this sense it is also interesting that the computer homology modeling of Vsp domes showed that the BtVsp2 dome seems to be the oddball among all the Vsp domes we studied (Figures 1 and 5). This is the largest in vivo study comparing the pathogenicity of individual serotypes of any RF spirochete. An intriguing observation from this structure/function study is that for many of the variables examined there was a good correlation between the Vsp dome homology dendrogram and the clinical manifestations of the infection and pathogen load in fluids and/or tissues. Probably the best example of this is that *B. hermsii* serotypes that clustered in the middle branch of the Vsp dome dendrogram, like Bh2 and Bh3, shared similar low systemic virulence and higher brain dissemination with Bt1, the one *B. turicatae* serotype we examined from the middle branch of the dendrogram. However, this study also has some important limitations, including that the analysis of serotypes was limited to 4 of the known Vsp serotypes of the HS1 strain of *B. hermsii*, that we did not study any of the Vlp serotypes, and that the clinical phenotype was studied in groups of only 4 SCID mice each.

The higher neuroinvasiveness of the 3 serotypes we examined from the middle branch of the Vsp dome dendrogram, Bh2, Bh3, and Bt1, relative to the three serotypes from the other two branches, Bh13, Bh27, and Bt2, suggests that there is something about the structure of the Vsp dome that may facilitate dissemination to the brain. However, confirmation of our findings can only be achieved via genetic manipulation of the expressed *vsp* gene in RF spirochetes, which is currently not possible. Another interesting observation is the lack of correlation between the intensity of peak bacteremia and the pathogen load in the brain (Figure 4), which has been observed before with Bt1 and Bt2. Because these results were obtained in SCID mice that are deficient in B and T cells we interpret this observation as evidence of differences in dissemination from blood to brain among serotypes and not simply as the result of niche selection in an immunoprivileged organ [22], [37]. These results are also consistent with our previous observation that some relapse serotypes of *B. hermsii* are found in blood but not brain during first relapse in immunocompetent *Balb/c* mice [22].

The computer homology modeling of Vsp domes revealed important differences in their polarity: although the overall Vsp structures were similar, the charge in the region that projects away from the spirochetes surface was very different between BtVsp2 and all the others (Figures 1 and 5). It is possible that polar amino-acids at the Vsp dome are involved in the interaction of RF spirochetes with eukaryotic cells. One possibility is that serotypes with less polar domes, and hence hydrophobic, like Bt2, would prefer interactions with proteins richer in non-polar amino acids such as those of collagenous tissues [38]. Consistent with this is the previous finding that Bt2 binds to glycosaminoglycans more efficiently than Bt1 and that recombinant BtVsp2, but not BtVsp1, binds to heparin and dermatan sulphate [39]. In the dendrogram, the BtVsp2 dome clustered next to the Vsp dome for the tick-associated protein, BhVsp33, also referred to as variable tick protein or Vtp [40]. It would be interesting to compare the Vsp dome structures of these 2 serotypes.

Bt2 originated from Bt1 through a gene conversion during chronic infection in one SCID mice that had been inoculated with tick homogenates responsible for an outbreak of tick-borne RF with prominent neuroborreliosis [8]. Despite an extensive interplasmidic duplication, the only detectable difference between Bt1 and Bt2 is whether their major expressed outer membrane lipoprotein is Vsp1 or Vsp2 [24]. Processed Vsp2 is distinguished from other Vsp and OspC proteins of Lyme disease spirochetes by a highly predicted isoelectric point [24]. Previously, circular dichroism spectra comparison of BtVsp1, Bt2Vsp2, BhVsp26, and *B. burgdorferi* OspC revealed similar, highly alpha-helical secondary structures in all of them [19]. Furthermore, all four proteins aggregated as dimers in solution and formed protease-resistant cores [19]. These observations indicate that Vsp and OspC proteins have a common compact fold and that their established functions are likely based on localized polymorphisms at their dome regions [21].

There are other known human pathogens that use variation of outer membrane proteins for modulation of tissue dissemination during infection. One example is the agent of malaria, *Plasmodium falciparum*, that sequesters infected erythrocytes in tissue microvessels that can be found in the brain, liver, lungs, kidneys, and other tissues; interestingly, antigenically different parasite populations exhibit different sequestration profiles [41]. Another example is *Neisseria gonorrhoeae*, where the pili responsible for adherence to different types of eukaryotic cells are composed of repeating pilin subunits; analysis of these pilin subunits has shown that there are constant, semivariable, and hypervariable regions and that hypervariable regions exhibiting differing amino acid compositions and isoelectric points result in variable adherence to different types of human cells [41]. In *B. burgdorferi*, the structures of OspC among tick populations are as diverse as the Vsp proteins in RF borrelias and could provide a method to modulate dissemination upon entering the mammalian host. Indeed, it has been demonstrated that OspC is required for mammalian infection but not for tick colonization, indicating a potential role for OspC in adapting the spirochete to the mammalian environment as well as conferring an ability to withstand a dramatic transition of environments [42]. Other studies that support a role for OspC in environmental adaptation showed, by directly injecting OspC mutants into host areas that normally become persistently infected, that the lack of fully formed OspC protein prevents establishment of infection [43], [44]. It has been speculated in the case of OspC that this role may be related to immune evasion, particularly the evasion of innate immunity that would normally destroy invading spirochetes before they have a chance to establish infection [44]. The fact that both Vsp and OspC expression is stopped via modulation of gene expression and not by plasmid loss [45] supports a model within the genus Borrelia for switching expression of outer membrane proteins to adapt to different environments. The findings from the present study extend the previous observations in *B. turicatae* to *B. hermsii* adding to the body of evidence suggesting that variation of outer membrane proteins in spirochetes may be an important determinant of the clinical manifestations of these infections.

## Supporting Information

Table S1 Multiple sequence alignment of relapsing fever Vsp's
(0.04 MB DOC)

Table S2 TaqMan PCR amplification of DNA from B. hermsii relapse mixa plasma, Borrelia turicatae serotype 1 (Bt1), and Borrelia hermsii serotype 21 (Bh21) DNA with TaqMan primers and probe sets for B. hermsii vsp genes 2, 3, 13, and 27, B. turicatae vsp genes
(0.03 MB DOC)

Table S3 TaqMan PCR amplification of the B. hermsii vsp genes 13 (vsp13) and 3 (vsp3) and the chromosomal house keeping gene 16S rRNA in blood and tissue samples from SCID mice inoculated with B. hermsii serotype 13
(0.03 MB DOC)
