# Supplementary material for: Structure-Function Investigation of Vsp Serotypes of the Spirochete Borrelia hermsii
Source: PLoS One. 2009 Oct 30;4(10):e7597. doi: 10.1371/journal.pone.0007597 (PMC2766631; doi:10.1371/journal.pone.0007597)
Supplement: Table S1 — Multiple sequence alignment of relapsing fever Vsp's (0.04 MB DOC) [file pone.0007597.s001.doc]

# Table S1. Multiple sequence alignment of relapsing fever Vsp's

<http://www.ebi.ac.uk/cgi-bin/clustalw> (CLUSTAL W 1.82)

MATURE LIPIDATED N-terminus

< signal peptide >↓<--likely disordered----><-----α1-------

O34000_BORTU MKRITLSALLMTLFL-LISCNNSGTSPKDGQAAKSDGTVIDLATITKNITDAVAFAKSVK 59

Q9Z6I0_BORTU MKRITLSALLMTLFL-LMSCNNSGTFPKDGQAAKSDGTVIDLATITKNIKDTVAFAKSVK 59

Q9RQ58_BORTU MKRITLSALLMTLFL-LMSCNNSGTSPKDGQAAKSDGTILDLATITKNIKDTVAFAKSVK 59

Q9RQ59_BORTU MKRITLSALLMTLFL-LMSCNNSGTSPKDGQAAKSDGTVIDLATISKNIKDTVAFAKSVK 59

Q45209_BORHE MRKR-ISAIIMTLFMVFMSCNNGGPELKSDEVAKSDGTVLDLAKVSKKIKEASAFAASVK 59

Q45215_BORHE MRKR-ISAIIMTLFMVFMSCNNGGPELKSDEVAKSDGTVLDLAKISKKIKDAVEFAASVK 59

Q45211_BORHE MRKR-ISAIIMTLFMVFMSCNNGGPELKSDEVAKSDGTVLDLAKISKKIKEASAFAASVK 59

P70896_BORHE MKKNTLSAILMTLFL-FISCNNGGPELKGNEVAKSDGTVLDLSKISSKIKNASAFAAGVK 59

VMP3_BORHE MRKR-ISAIIMTLFMVFMSCNNGGPELKSDEVAKSDGTVLDLAKISKKIKDASDFAASVK 59

Q45212_BORHE MRKR-ISAIIMTLFMVFMSCNNGGPELKSDEVAKSDGTVLDLAKISKKIKDAVEFAASVK 59

VMP24_BORHE MRKR-ISAIIMTLFMVFMSCNNGGPELKSDEVAKSDGTVLDLAKVSKKIKEASAFAASVK 59

Q45207_BORHE MRKR-ISAIIMTLFMVLVSCNNGGPKLKSDEVAKSDGTVLALAKISKKIKDASDFATSVK 59

Q45214_BORHE MRKR-ISAIIMTLFMVFMSCNNGGPELKSDEVAKSDGTVLDLAKVSKKIKEASAFAASVK 59

Q45208_BORHE MRKR-ISAIIMTLFMVFMSCNNGGPELKSDEVAKSDGTVLDLAKISAKIKEASAFAASVK 59

Q45213_BORHE MKKNTLSAILMTLFL-FISCNNGGPELKKGEVTKSDGTVLDLSKISANIKNAVTFAASVQ 59

Q45206_BORHE MRKR-ISAIIMTLFMVFMSCNNGGPELKSDEVAKSDGTVLDLAKVSKKIKEVSAFAASVK 59

Q53329_BORHE MRKR-ISAIIMTLFMVFMSCNNGGPELKSDEVAKSDGTVLDLAKISAKIKEASAFAASVK 59

Q45210_BORHE MRKR-ISAIIMTLFMVFMSCNNGGPELKSDEVAKSDGTVLDLAKISKKIKEASAFAASVK 59

*:: :**::****: ::****.*. * .:.:*****:: *:.:: :*.:. ** .*:

<variable region 1> < VR2

---α1--------> <β1> <β2> <---α2-----------

O34000_BORTU DVHTLVKSIDELAKAIGKKIGANGLE--TDAD---KNAKLISGAYSVISAVDTKLA 110

Q9Z6I0_BORTU EVHTLVKSIDELAKAIKKKIQAGGLQ--DDTDN--LNGTLLAGAYQIMADADSKLT 111

Q9RQ58_BORTU EVHTLVKSIDELAKAIGKKIQNNGTL-TDDGS-TDKNTSLMSGVYSIVLDIDKKSK 113

Q9RQ59_BORTU DVHTLVKSIDELAKAIGQKIQQNSDQFANDGA---HNGSLISGAFQVILTVETKLK 112

Q45209_BORHE EVETLVKSVDELAKAIGKKIKNDGT-LEFADADKDKNGSLIAGAFQIILIAEGKLK 114

Q45215_BORHE EIETLVKSIDELAKAIGKKIKQNSEDLEVDNGKNNKNGELVAGAFQVMLTVKAKLE 115

Q45211_BORHE EIETLVKSIDELAKAIGKKIKNDGT-LEAIAD---KNGSLIAGVVSVAEAVEKKLG 111

P70896_BORHE EIHALVKSVGEFAKGIGNKVTQNTGVIDADAGG-NNNGQIIVGAYSLISGLKTQVE 114

VMP3_BORHE EVHTLVKSIDELAKAIGKKIKNDNSNFEDEND---HNGSLIAGVFQVILTVKAKLT 112

Q45212_BORHE EIETLVKSIDELAKTIGQKLTKDTGVLAAD--ANNNNGGLIAGVYGIVTDVGTKLD 113

VMP24_BORHE EVETLVKSVDELAKAIGKKIKNDGG-LDTEAG---QNGSLIAGVHSVVSAVKIKVG 111

Q45207_BORHE EVHTLVKSIDELAKAIGKKIHNDGS-LTTEDG---KNGSLLAGVHSVISAVKTKLG 111

Q45214_BORHE EVETLVKSVDELAKAIGKKIKNDDDGFDTEAN---KNGSLLAGTLQLMFAVGTKLE 112

Q45208_BORHE EVHTLVKSVDTLAGAIGKKIKSDG-KFDAMAG---KNGSLLAGAYNVALDINSKLT 111

Q45213_BORHE EVETLVKSIDELAKAIGQKVNADGL----TAEA-NKNDSLVAGVYQLISDVQGKLT 110

Q45206_BORHE EVHTLIKSIGDLAKAIGKKIKTDETGTLESS-TADQNEQLVAGAFQVVSTVKGELE 114

Q53329_BORHE EVQTLVKSVDTLAGAIGKKIKSDGK-FDAMAG---KNGSLIAGAYNVALDINSKLT 111

Q45210_BORHE EVHALVRSIDDIAKGIKKKIAANGL--EDDANGSNHHTPLMAGVFSVATTIEKKSG 113

::.:*::*:. :* * :*: :: *. : :

VR2 > < VR3 > < VR4

-α2> <----α3------------------> <-α4->

O34000_BORTU SLEKKVGISD-DLKGKITTVKNASTSFLTKAKSKTADLGKD--DVKDADAKTAIDIADTG 167

Q9Z6I0_BORTU ALEGKSEKFA-GIKDKVISAKQKSTAFLNKLKSENATLGAASAAVSSANAKEAIDRNN-A 169

Q9RQ58_BORTU ALSVLESFKEQILDEKIISFTTATKAFLDKLKSKHAELGVDQGAATKDNAQKAIDRVNKA 173

Q9RQ59_BORTU SLEDTVGLSD-TLKTKVTSSKIASRAFLDKVKSKHTELGKE--GASDADAKAAILVSN-G 168

Q45209_BORHE GLDK-EAGISEALKAKVTDAEAKSKAFLAKVKGQTATLGKND--ASDDDTKKAIKKDNSD 171

Q45215_BORHE KLGN-TPEISEELKGKITDSKSKCKEFVDKVKADSDISKAEA---TDEHVKKAIDQVNAP 171

Q45211_BORHE ELQ--VAGFLKGLNEKVQDVDAKVKAFTKKLKDKHVVLGAADGATTDDNAKKAIDRVNQV 169

P70896_BORHE ELGK-KDGISDGLKEKLDDVSKKGTAFLDKVKADAELCKKDV---TDENAKKALDVNNAS 170

VMP3_BORHE SLEQ-IIGISDELKTEVGMVKKESEAFVTQVKSKHTDLAKEG--VTDAHAKSAILVTDGT 169

Q45212_BORHE GLLK-VNGISEDIKTKINDSKSKGTAFLSKVKGDDDLCKKDA---TDAHAKNAIDKNDNT 169

VMP24_BORHE ALET-TSGISNELKTKITEVKSKAEAFLNKLKDGHTELGKKD--ASDDDTKKAIKKDNSD 168

Q45207_BORHE SLEQKAIGEFAGMKVQVVAIKTASIDLLNKFKDKNAELGKNE--VSNDDAKAAILVSNTT 169

Q45214_BORHE SLEK-IAGISDEVRGKVIVVKTENTALITKLKGGDASLGKND--ASDSDAKNAIDKSDVT 169

Q45208_BORHE VLDG-KAGLSSLLKAKVTAAKTSGESFSNKLKTEHTDLGKEE--ASDDNAKAALLVTNAT 168

Q45213_BORHE KLEI-GASKFAGLKEKVVAAKKGSDDFLTKVKAQHNNLG------QSAEAPKAIKKGNAD 163

Q45206_BORHE SLVQ-VDGISDDLKAKVNEAKNANDGLLSKFKSSAKDNESVK---KDEEAKKVIDRTN-- 168

Q53329_BORHE VLDG-KAGLSSLLKAKVTAAKTSGESFSNKLKTEHTDLGKEE--ASDDNAKAALLVTNAT 168

Q45210_BORHE ELQ--VAQSLKNLGEKVKDVEAKAKAFIIKLKNQHATLGAADGAATDANARNAIDKSDAT 171

* : :: : : * . .. .: :

VR4 >

<---------α5------------------><-disordered>

O34000_BORTU AKDKGAEELIKLNTAIDALLTSAEAAVTAAINALS-TPAKSASTVQSN------------ 214

Q9Z6I0_BORTU TKTKGAKELEELNTAIDTLLKAAEDAVTAAINALS-TPAKSASTAQSN------------ 216

Q9RQ58_BORTU DGENGAKELGELNTAVDALLKAAEATVTSAINALS-TPAKSESTKPSNT----------- 221

Q9RQ59_BORTU TKDKGVDELVKLNTEIDALLTAAEAAVTAAINALS-TPAKSDAPAQSN------------ 215

Q45209_BORHE -KTKGASELEALNTAVDALLKAAEGEVEAAIKELT-APVKAEKPSQNN------------ 217

Q45215_BORHE AGEKGVVELVKLNKSIGELLKAANAAAEAAIAELT-APVKAEKPSQNN------------ 218

Q45211_BORHE NGENGAEELGKLNTAVDALLKAAEGEVEAAITELT-VPVKVEKPSQNN------------ 216

P70896_BORHE -KEKGAKELGDLNTAVDGLLTAANGEVEAAIKELTAAPAKPATPVKP------------- 216

VMP3_BORHE -KDKGAAELIKLNTAIDELLKAANDAVETVIKELT-ASVKAEKPSQNN------------ 215

Q45212_BORHE -GGKGKTELIALNTAIDELLKAANEAVEAAIKELT-APVKAEKPSQNN------------ 215

VMP24_BORHE -KTKGASELEALNTAVDALLKAAEGEVEAAIKELT-APVKAEKPSQNN------------ 214

Q45207_BORHE -KDKGASELEALNTAIDGLLKAANGAVEAAITELT-VPVKVEKPSQNN------------ 215

Q45214_BORHE -GGKVRKSLFKLNTAVDALLKAAEGEVEAAIKELT-APVKVEKPSQNN------------ 215

Q45208_BORHE -KNKGVTELEALNTAVDALLKAAEGEVEAAIKELT-APVKVEKPSQNN------------ 214

Q45213_BORHE -STKGAEELGKLNTAIDELLTAAKDAVEAAIADVTAAPAKPATPVKP------------- 209

Q45206_BORHE ---ASATELKKLDTAVDELLKAANEAVSAAIAELT-APAKS------------------- 205

Q53329_BORHE -KNKGVTELEALNTAVDALLKAAEGEVEAAIKELT-APVKVEKPSQNN------------ 214

Q45210_BORHE -GGKGKEELIALNTAIDGLLKAAEGEVEAAIKELT-APVKVEKPSQNN------------ 217

.* *:. :. **.:*: . :.* :: ...*

KEY to alignment

| UniProt ID* common name prior names used |
| --- |
| O34000_BORTU Vsp1 VspA, VmpA |
| Q9Z6I0_BORTU Vsp2 VspB, VmpB |
| Q9RQ58_BORTU Vsp6 VspF |
| Q9RQ59_BORTU Vsp5 VspE |
| Q45209_BORHE Vsp22 Vmp22 |
| Q45215_BORHE Vsp8 Vmp8 |
| Q45211_BORHE Vsp26 Vmp26 |
| P70896_BORHE Vsp |
| VMP3_BORHE Vsp3 Vmp3 |
| Q45212_BORHE Vsp2 |
| VMP24_BORHE Vsp24 Vmp24 |
| Q45207_BORHE Vsp13 Vmp13 |
| Q45214_BORHE Vsp6 Vmp6 |
| Q45208_BORHE Vsp1 Vmp1 |
| Q45213_BORHE Vsp33 Vmp33 |
| Q45206_BORHE Vsp11 Vmp11 |
| Q53329_BORHE Vsp Vmp |
| Q45210_BORHE Vsp27 Vmp27 |

*BORTU: *Borrelia turicatae*; BORHE: *Borrelia hermsii*
