# Supplementary material for: Structure-Function Investigation of Vsp Serotypes of the Spirochete Borrelia hermsii
Source: PLoS One. 2009 Oct 30;4(10):e7597. doi: 10.1371/journal.pone.0007597 (PMC2766631; doi:10.1371/journal.pone.0007597)
Supplement: Table S2 — TaqMan PCR amplification of DNA from B. hermsii relapse mixa plasma, Borrelia turicatae serotype 1 (Bt1), and Borrelia hermsii serotype 21 (Bh21) DNA with TaqMan primers and probe sets for B. hermsii vsp genes 2, 3, 13, and 27, B. turicatae vsp genes (0.03 MB DOC) [file pone.0007597.s002.doc]

| **Table S2.** TaqMan PCR amplification of DNA from *B. hermsii* relapse mix^a^ plasma, *Borrelia turicatae* serotype 1 (Bt1), and *Borrelia hermsii* serotype 21 (Bh21) with TaqMan primers and probe sets for *B. hermsii* *vsp* genes 2, 3, 13, and 27, *B. turicatae* *vsp* genes 1 and 2, and the *Borreliae* chromosomal gene 16S rRNA | | | | | | | |
| --- | --- | --- | --- | --- | --- | --- | --- |
|  | Gene | | | | | | |
| DNA sample | *Bhvsp2* | *Bhvsp3* | *Bhvsp13* | *Bhvsp27* | *Btvsp1* | *Btvsp2* | *16S rRNA* |
| Relapse mix | 25.26^b^ | 21.18 | 22.4 | 23.22 | >40^c^ | >40 | 22.89 |
| Cultured Bt1 | >40 | >40 | >40 | >40 | 28.7 | >40 | 31.57 |
| Cultured Bh21 | 15.18 | 15.55 | 14.32 | 14.87 | 16.29 ^d^ | >40 | 17.18 |
| ^a^ Relapse mix plasma was produced by 5 serial passages of *B. hermsii* strain HS1 serotypes into *Balb/c* mice followed by amplification to peak bacteremia in SCID mice (see methods for details). | | | | | | | |
| ^b^ Results are shown as Ct values (threshold of amplification) | | | | | | | |
| ^c^ Not detectable (Ct>40). | | | | | | | |
| ^d^ Needs to be confirmed. | | | | | | | |
