# Supplementary material for: Structure-Function Investigation of Vsp Serotypes of the Spirochete Borrelia hermsii
Source: PLoS One. 2009 Oct 30;4(10):e7597. doi: 10.1371/journal.pone.0007597 (PMC2766631; doi:10.1371/journal.pone.0007597)
Supplement: Table S3 — TaqMan PCR amplification of the B. hermsii vsp genes 13 (vsp13) and 3 (vsp3) and the chromosomal house keeping gene 16S rRNA in blood and tissue samples from SCID mice inoculated with B. hermsii serotype 13 (0.03 MB DOC) [file pone.0007597.s003.doc]

| **Table S3.** TaqMan PCR amplification of *B. hermsii* *vsp* genes 13 (*vsp13*) and 3 (*vsp3)* and chromosomal gene *16S rRNA* in blood and tissue samples from SCID mice that had been inoculated with *B. hermsii* serotype 13 | | | | | | |
| --- | --- | --- | --- | --- | --- | --- |
| Mouse number | Sample | *vsp13*^a^ | *vsp3* | *vsp3*-*vsp13*^b^ | *16S rRNA* | *vsp13-16S rRNA*^c^ |
| 1 | blood culture | 26.86 | 27.83 | 0.97 | 26.62 | 0.23 |
|  | spleen | 28.81 | 29.29 | 0.484 | 28.29 | 0.52 |
| 2 | plasma | 32.73 | 33.99 | 1.26 | 32.97 | -0.24 |
|  | spleen | 23.27 | 24.47 | 1.19 | 22.87 | 0.397 |
| ^a^ Results are shown as Ct values (threshold of amplification). | | | | | | |
| ^b^ The mean (SD) Ct for *vsp3*-*vsp13* was 0.97 (0.35). | | | | | | |
| ^c^ The mean (SD) Ct for *vsp13*-*16S rRNA* was 0.23 (0.33). | | | | | | |
